# Workplace violence and fear of violence: an assessment of prevalence across industrial sectors and its mental health effects

**DOI:** 10.5271/sjweh.4230

**Published:** 2025-09-01

**Authors:** Vanessa Gash, Niels Blom

**Affiliations:** 1 City St Georges, University of London, London, UK.; 2 University of Manchester, Manchester, UK.

**Keywords:** common mental disorder, longitudinal study, workplace harassment, workplace assault

## Abstract

**Objectives:**

This study aimed to (i) examine variance in the prevalence of workplace violence and fear of violence in the United Kingdom by industrial sector and (ii) determine the mental health effects thereof using longitudinal data.

**Methods:**

We used the United Kingdom Household Panel Study (UKHLS), a nationally representative survey with mental health indicators collected annually allowing us to determine common mental disorders (CMD) at baseline, one year prior and one year later. Using weighted logistic regression and lagged dependent variable regression, we examined prevalence of violence and fear of violence by sector and the effect of violence on CMD risk. We supplemented our analyses with the views of those with lived experience.

**Results:**

Workers employed in public administration and facilities had the highest risks of workplace violence, with predicted probabilities (PP) of 0.138 [95% confidence interval (CI) 0.116–0.160], and these were not statistically different from the second highest sector of health, residential care, and social work (PP 0.118, 95% CI 0.103–0.133). Workplace violence increased CMD risk [adjusted odds ratio (OR_adj_) 1.400, 95% CI 1.182–1.658] as did fear of violence at work (OR_adj_ 2.103, 95% CI 1.779–2.487), adjusting for prior CMD. Moreover, the effect of violence and fear of violence on CMD remained when we investigated CMD one year later.

**Conclusions:**

A high prevalence of workplace violence and fear of workplace violence was found in multiple different industrial sectors – >1 in 10 workers were exposed to violence in the last 12 months in 30% of sectors and >1 in 20 workers were exposed in 70% of sectors. Both violence and fear of violence were associated with enhanced CMD risk at baseline and one year later.

Workplace violence is a significant problem with under-examined productivity effects. In a global survey, just under 1 in 5 workers reported exposure to psychological violence and harassment at work, and 1 in 10 reported exposure to physical violence during their working-lives (1). In the EU, 13% of workers were exposed to adverse social behavior at work (encompassing verbal abuse, threats, bullying, harassment or violence) in the past year (2). In the UK, the Health and Safety Executive (the regulator for workplace health and safety) found 1% of all adults of working age, in the 12 months prior, experienced a physical assault or threat of assault at work, a rate found to be consistent across the time periods examined (3). Prevalence of workplace bullying or harassment is much higher in the UK, with 1 in 10 workers exposed to it in the 12 months prior (4). Workplace violence covers a broad range of adverse social interactions and behaviors committed by or towards employees. It includes encounters between colleagues and between workers and service users (5). It can also include incidents of domestic abuse experienced at work, with abusers known to pursue victims in the workplace (6). Direct and indirect exposure to violent acts or threats of violence at work can be anticipated to lead to anxiety (7) and fear of further victimization (8). Workplace violence, especially when persistent, may cause psychological disorders including common mental disorders (CMD) of generalized anxiety and depression. Post-traumatic stress disorders may arise among workers who feel unable to leave a violent workplace to avoid or protect themselves from future exposure to workplace violence, with the disorder understood to be triggered by both multiple chronic traumas as well as one extreme event (8). This dynamic may be compounded by fears surrounding job change (9) with research tending to find intentions to quit to be much higher than the number of workers who leave their job as a result of workplace violence (10). While some have found workers in the human service industry, with frequent client interactions, to partially acclimatize themselves to workplace violence given high prevalence rates (5), there is a notable body of work that confirms the strong association between workplace violence and psychological harms, including CMD risk, depression and post-traumatic stress disorder (PTSD) (4) (10) (11) alongside increased suicide risk (12). Though some of this research includes analyses of prospective data (13), few are based on prospective national samples with some exceptions (12) (14). Therefore, one of our key contributions is an analysis of the prospective association between workplace violence and poor mental health among a nationally representative sample using direct measures of workplace violence and fear of violence. Moreover our longitudinal data allows for controls of causal direction with poor mental health known to be predictive of exposure to workplace violence (15) (16).

There has been substantial policy attention to the costs of workplace violence on employee well-being though the field is characterized by analyses on sectoral sub-samples (17) (18) (19). Partly this reflects the unique nature of violence risk for different occupational groups, with healthcare professionals (10) and those who work with troubled service users (20) (21) expected to be more prone. Additionally, it reflects sectoral variance in the management of workplace violence, with healthcare more proactive given its problems with job retention (22). This is important as it may be that sectors less exposed to skill gaps in recruitment also face high rates of workplace violence of which we are less aware. Sectorial specificity in academic work on the topic also risks fragmenting knowledge of workplace violence into occupational or sectoral silos. Therefore, another aim of this paper is to provide estimates of industry and occupational gradient-specific prevalence of workplace violence.

Another contribution concerns our analysis of a data infrequently examined on the topic: the United Kingdom Household Panel Study (UKHLS), a nationally representative panel survey of approximately 40 000 households (23). Official estimates of workplace violence for the UK use the Crime Survey for England and Wales (CSEW), yet this data offers low prevalence rates of 1% compared to those found in other advanced economies. For example, a representative sample of Danish employees established a prevalence of 8% using a similar definition of exposure to physical assault or threats of assault in the 12 months prior (18). There is also utility in using the UKHLS given that it has recently been validated and cross-checked against the CSEW for the analysis of violence (24).

## Methods

### Study design

We used waves 10, 11, and 12 of the UKHLS. Data collection for each wave spans a two-year period and for our analyses covered 2018–2022. Many indicators are collected annually, via face-to face interview, including indices relating to mental health and labor market status, allowing for assessments of changes in mental health over time. However, the violence indicators have only been asked once of the entire sample, in wave 11, and it is for this reason that we limit our analyses to wave 11 and to those waves adjacent to it. It should also be noted that while a portion of data collection for wave 11 coincided with the COVID-19 pandemic, data collection did not stop as a result of it with interviews conducted online or by telephone from March 2020 to April 2022 to ensure continuity in the panel design, with 32.5% of the sample collected after the March 2020 UK national lockdown. We therefore conducted tests of a possible COVID-19 effect by de-selecting respondents who completed interviews during lockdown, which led to the same conclusions.

### Measures of violence and fear of violence

We generated an amalgamate category of workplace violence, which included those who have been ‘physically attacked’ and/or ‘insulted, threatened or shouted at’ in the 12 months prior. The precise wording of the questionnaire asked: “In the last 12 months, have you been insulted, called names, threatened or shouted at, in any of these places? If so, which ones?”, and “In the last 12 months, have you been physically attacked in any of these places? If so, which ones?”. We combined both questions given low prevalence of being attacked. We determined fear of violence from positive responses to a question that asked: “In the last 12 months, have you felt unsafe in any of these places?”. For each of the above three questions, respondents were provided with a list of 11 locations and could select multiple, and we selected those who reported the workplace as the location of their exposure to violence or fear of violence. Our measure of fear of violence was unlike more widely used measures that measure fear of crime as feeling unsafe in one’s neighborhood at night-time (25). So, for our purposes, the data were better suited for an analysis of fear of violence at work. We could not, however, examine the perpetrators of violence using these data.

### Measures of mental health

Associations between violence and CMD were examined using an aggregate dichotomous threshold variable based on the general health questionnaire (GHQ-12) (26). Construct validity of GHQ-12 against psychiatric diagnosis based on clinical interview of anxiety and depression have established strong overlap (27). The battery collects indicators on current mental health, with respondents asked to reflect on how they have felt over the past few weeks. The threshold variable we applied is widely used in the literature (28) and identifies those with clinically significant mental disorders. The variable scores 1 to those reporting positively to poor mental health indicators: for those with responses “rather more than usual” and “much more than usual”, while those who reported “not at all” and “no more than usual”, scored 0. The sum of the index ranges from 0–12, and the threshold variable groups those with an index of ≥4 as suffering from a CMD (26).

### Measures of industrial sector

Industrial sector and occupational status, which we introduced as an important confounder of violence risk, were generated post-interview using respondents’ job description. We used the two-digit Standard Industrial Classification (SIC) 2007 to examine industrial sector and applied the simplified 10 categorical classification. Due to sample size, we excluded the armed forces and combined skilled agricultural occupations with craft and related trades resulting in 8 occupational categories (see table 1).

**Table 1:** Unweighted descriptive statistics (percentages are weighted). [CMD=common mental disorder; SD=standard deviation]

		Sample 1				Sample 2		
		N=11 366				N=9908		
		N	%	Mean (SD)		N	%	Mean (SD)
CMD at t+1 (yes)						2097	22.2	
CMD at t (yes)		2197	20.3					
CMD at t-1 (yes)		1938	17.3			1683	17.3	
Violence (yes)		914	8.3			791	8.2	
Fear of violence (yes)		805	7.7			682	7.5	
Industrial classification								
	Public administration & facilities	1086	9.0			998	9.6	
	Health, residential care & social work	2167	17.7			1889	17.7	
	Education	1534	13.2			1353	13.4	
	Wholesale & retail	1306	12.4			1131	12.3	
	Transportation & storage	498	4.3			413	4.1	
	Manufacturing & construction	1647	15.3			1393	14.6	
	Information, communication, finance & insurance	861	7.4			775	7.7	
	Business administration & support services	1341	12.2			1177	12.2	
	Arts, entertainment and other services	567	5.3			490	5.3	
	Accommodation & food services	359	3.3			289	3.1	
Occupational classification								
	Legislators, senior officials & managers	1767	15.4			1544	15.6	
	Professionals	2023	16.9			1809	17.4	
	Technicians & associate professionals	2096	18.0			1881	18.6	
	Clerks	1486	12.7			1308	12.9	
	Service workers & shop & market sales	1846	16.3			1570	15.8	
	Agricultural, fishery, craft, and related trades	692	6.6			584	6.2	
	Plant & machine operators & assemblers	583	5.3			477	5.1	
	Elementary occupations	873	8.7			735	8.5	
Contract type								
	Permanent	10550	92.8			9191	92.7	
	Temporary	816	7.2			717	7.3	
Work hours								
	Fulltime	8395	73.6			7287	73.4	
	Parttime	2971	26.4			2621	26.7	
Working time								
	Standard	7472	63.9			6591	64.9	
	Non-standard	3894	36.1			3317	35.1	
Gender								
	Men	5136	48.0			4424	47.4	
	Women	6230	52.0			5484	52.6	
Ethnicity								
	White	9839	92.3			8683	92.8	
	Mixed, multiple or other	282	2.0			245	2.1	
	Asian	879	3.8			698	3.5	
	Black	366	1.9			282	1.7	
Age range (17–85)				45.7(12.5)				46.1(14.4)
Work autonomy range (0–4)				2.1(0.8)				2.1(0.8)

### Confounders

Not all workplaces, or jobs held within them, are equal, and prior research has found certain features of the workplace to be confounders of workplace violence and of mental health (2) (21) (29) (9). We therefore introduced the following variables to improve estimate precision of our key covariates. Occupational status is an important predictor of violence, with those from lower status groups often found to be more exposed to violence risk (30). We therefore adjusted for occupational status and used the International Classification of Occupations 1988 (ISCO 88). We distinguished between those on permanent versus fixed-term or temporary contracts as job insecurity is known to be predictive of both poor mental health (31) and enhanced violence risk (32). We adjusted for working-time, distinguishing between full- and part-time workers, and adopted a cut-off of ≥30 hours per week. We adjusted for job autonomy and standard versus nonstandard working time, which were asked in wave 10 and fed forward. The job autonomy scale was constructed using responses relating to control over task performance, pace, manner, and order, as well as work hours, with answer categories ranging from (0) none to (3) a lot (Cronbach’s alpha=0.855). Non-standard working was defined as working most weekends, usually working nights, evenings, rotating shifts or with varying patterns. We further distinguished variance in risk by age (continuous), sex (men or women), and ethnicity (distinguishing between White (British), Asian (British), Black (British), and mixed/multiple or other ethnic backgrounds).

### Statistical method

We examined two longitudinal samples, the first (N=11 366) examined the prevalence of workplace violence across industrial sectors as well as the association between workplace violence on CMD risk adjusting for CMD at t-1, with CMD one year prior correlated with current CMD risk. The second sample (N=9908) examined whether the effects of workplace violence are persistent, presenting tests of the association between workplace violence and CMD at t+1. All analyses select on employees as key controls were not asked of the self-employed, and we further excluded those with missing values.

**Figure 1:**
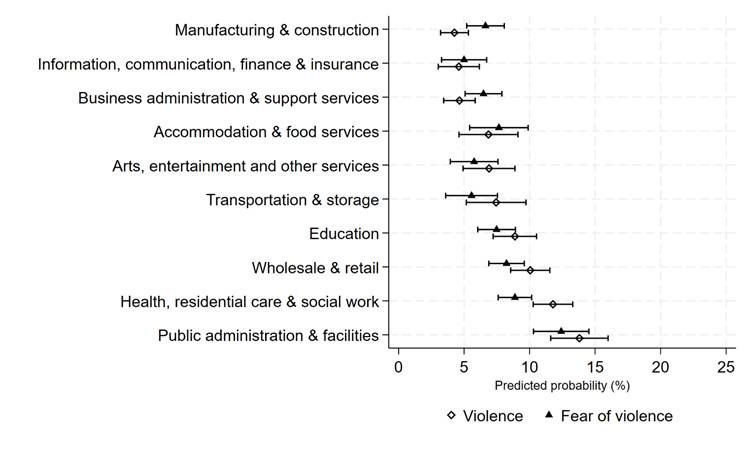
Prevalence of workplace violence and fear of violence at work by industrial sector. Note: Weighted estimates derived from average marginal effects based on adjusted models shown in table 2 (N=11 366), which adjust for occupational status, contract-type, work hours, working time, work autonomy, sex, age, and ethnicity. Predicted probabilities and pair-wise comparisons are presented in supplementary table S1.

Table 1 presents unweighted descriptive statistics for both samples. Variance in the probabilities of workplace violence and fear of violence by industrial sector are presented in table 2 for sample 1, with adjusted and unadjusted odds ratios (OR) derived from weighted bivariate and logistic regression analyses. Figure 1 provides a graphic representation of violence prevalence by sector using average marginal effects based on the models presented in table 2. Pair-wise comparisons of these average marginal effects which show statistically significant differences in workplace violence by sector are presented in supplementary material, www.sjweh.fi/article/4230, table S1.

**Table 2:** Results from the logistic regression analysis, showing the risk of workplace violence and fear of violence by industrial classification, N=11 366. [CI=confidence interval; OR=odds ratio; OR_adj_=adjusted odds ratio]

		Number of observations	Observations violence (%) ^a^	Violence	
				OR (95% CI)	OR_adj_ (95% CI) ^b^
Industrial classification					
	Public administration & facilities	1086	125 (12.9)	1	1
	Health, residential care & social work	2167	262 (12.3)	0.944 (0.753–1.183)	0.828 (0.651–1.054)
	Education	1534	118 (7.9)	0.579 (0.446–0.752)	0.597 (0.451–0.792)
	Wholesale & retail	1306	146 (12.1)	0.926 (0.726–1.180)	0.688 (0.530–0.894)
	Transportation & storage	498	43 (9.3)	0.692 (0.485–0.989)	0.491 (0.330–0.731)
	Manufacturing & construction	1647	62 (4.0)	0.280 (0.207–0.379)	0.268 (0.192–0.374)
	Information, communication, finance & insurance	861	37 (3.8)	0.262 (0.176–0.390)	0.291 (0.194–0.436)
	Business administration & support services	1341	54 (3.9)	0.276 (0.199–0.383)	0.294 (0.210–0.411)
	Arts, entertainment and other services	567	37 (7.2)	0.521 (0.364–0.745)	0.451 (0.312–0.654)
	Accommodation & food services	359	30 (9.4)	0.695 (0.470–1.026)	0.448 (0.296–0.678)
		Number of observations	Observations fear of violence (%) ^a^	Fear of violence	
				OR (95% CI)	OR_adj_ (95% CI) ^b^
Industrial classification					
	Public administration & facilities	1086	113 (11.2)	1	1
	Health, residential care & social work	2167	208 (9.6)	0.842 (0.659–1.075)	0.678 (0.524–0.878)
	Education	1534	115 (7.4)	0.634 (0.482–0.833)	0.559 (0.418–0.749)
	Wholesale & retail	1306	119 (10.2)	0.895 (0.690–1.160)	0.623 (0.471–0.824)
	Transportation & storage	498	33 (6.7)	0.564 (0.375–0.846)	0.405 (0.261–0.629)
	Manufacturing & construction	1647	70 (5.4)	0.447 (0.336–0.595)	0.490 (0.357–0.673)
	Information, communication, finance & insurance	861	30 (3.6)	0.299 (0.198–0.450)	0.360 (0.238–0.546)
	Business administration & support services	1341	55 (5.6)	0.467 (0.346–0.631)	0.478 (0.350–0.651)
	Arts, entertainment and other services	567	24 (6.0)	0.505 (0.343–0.744)	0.421 (0.282–0.627)
	Accommodation & food services	359	38 (12.3)	1.103 (0.768–1.585)	0.574 (0.389–0.848)

^a^ Percentages are based on weighted sample. ^b^ Adjusted for occupational classification, contract type, work hours, working time, work autonomy, gender, age, and ethnicity. Full tables available in supplementary tables S3 and S4.

Table 3 applies lagged-dependent variable regression models (33), which include estimates of our dependent variable at t-1. These models allow us to control for CMD one year prior to approximate a causal measure of the effects of violence on well-being, with prior mental health conditions likely to be predictive of current mental health. Table 4 examines whether the effects of workplace violence persist over time through an assessment of workplace violence at t on CMD risk at t+1. We provide sensitivity tests to determine whether a linear specification of CMD GHQ-12 led to similar conclusions in appendix table 6. All analyses are weighted to account for the complex sample design, unequal selection probabilities, and non-response/attrition as is advised for these data (34).

**Table 3:** Results from the logistic regression analysis, showing the showing the risk of having common mental disorders (GHQ-12) at time t by workplace violence, fear of violence at work, and industrial classification, N=11 366. [CI=confidence interval; OR=odds ratio; OR_adj_=adjusted odds ratio].

		CMD at t			
		Number of observations	Observations with CMD at t (%) ^a^	OR (95% CI)	OR_adj_ (95% CI) ^b^
Common mental disorders at t-1					
	No	9428	1291 (14.5)	1	1
	Yes	1938	906 (48.0)	5.429 (4.886–6.032)	4.861 (4.363–5.417)
Violence at work					
	No	10 452	1919 (19.1)	1	1
	Yes	914	278 (34.1)	2.201 (1.908–2.538)	1.400 (1.182–1.658)
Fear of violence at work					
	No	10 561	1885 (18.7)	1	1
	Yes	805	312 (39.4)	2.817 (2.439–3.253)	2.103 (1.779–2.487)
Industrial classification					
	Public administration & facilities	1086	224 (23.0)	1	1
	Health, residential care & social work	2167	482 (22.7)	0.984 (0.823–1.177)	0.902 (0.741–1.099)
	Education	1534	331 (22.2)	0.956 (0.790–1.156)	0.941 (0.762–1.164)
	Wholesale & retail	1306	246 (20.8)	0.877 (0.722–1.065)	0.913 (0.736–1.134)
	Transportation & storage	498	73 (13.7)	0.532 (0.396–0.716)	0.797 (0.573–1.108)
	Manufacturing & construction	1647	226 (14.8)	0.581 (0.477–0.707)	0.777 (0.622–0.971)
	Information, communication, finance & insurance	861	166 (20.8)	0.880 (0.706–1.098)	0.988 (0.778–1.254)
	Business administration & support services	1341	249 (20.7)	0.872 (0.717–1.060)	1.024 (0.829–1.265)
	Arts, entertainment and other services	567	112 (20.9)	0.883 (0.692–1.127)	0.923 (0.707–1.204)
	Accommodation & food services	359	88 (22.0)	0.946 (0.713–1.256)	0.792 (0.575–1.091)

^a^ Percentages are based on weighted sample. ^b^ Adjusted for occupational classification, contract type, work hours, working time, work autonomy, gender, age, and ethnicity.

**Table 4:** Results from the logistic regression analysis, showing the risk of having common mental disorders (GHQ-12) one year later (t+1) by workplace violence, fear of violence at work, and industrial classification, N=9908. [CI=confidence interval; OR_adj_=adjusted odds ratio].

		CMD at t +1			
		Number of observations	Observations with CMD at t+1 (%) ^a^	OR (95% CI)	OR_adj_ (95% CI) ^b^
Common mental disorders at t-1					
	No	8225	1322 (17.1)	1	1
	Yes	1683	775 (47.0)	4.310 (3.859–4.814)	3.775 (3.368–4.231)
Attacked and/or insulted/threatened at work					
	No	9117	1835 (21.0)	1	1
	Yes	791	262 (36.5)	2.172 (1.868–2.525)	1.461 (1.226–1.742)
Felt unsafe at work					
	No	9226	1852 (20.8)	1	1
	Yes	682	245 (39.7)	2.508 (2.149–2.928)	1.731 (1.450–2.068)
Industrial classification					
	Public administration & facilities	998	242 (27.2)	1	1
	Health, residential care & social work	1889	435 (23.0)	0.803 (0.671–0.963)	0.729 (0.599–0.886)
	Education	1353	318 (24.6)	0.873 (0.722–1.055)	0.848 (0.688–1.045)
	Wholesale & retail	1131	227 (22.8)	0.794 (0.653–0.965)	0.806 (0.650–0.999)
	Transportation & storage	413	70 (16.9)	0.547 (0.407–0.735)	0.816 (0.590–1.128)
	Manufacturing & construction	1393	220 (16.4)	0.528 (0.433–0.644)	0.722 (0.579–0.901)
	Information, communication, finance & insurance	775	145 (18.4)	0.605 (0.480–0.763)	0.652 (0.510–0.833)
	Business administration & support services	1177	245 (22.4)	0.774 (0.636–0.942)	0.849 (0.689–1.047)
	Arts, entertainment and other services	490	116 (24.2)	0.857 (0.671–1.096)	0.884 (0.679–1.151)
	Accommodation & food services	289	79 (30.0)	1.150 (0.869–1.523)	0.927 (0.677–1.270)

^a^ Percentages are based on weighted sample. ^b^ Adjusted for occupational classification, contract type, work hours, working time, work autonomy, gender, age, and ethnicity.

### Lived experience engagement

Our statistical analyses are supplemented with the views of those with lived experience of violence and abuse in accordance with the aims of our research program. Participants’ views were obtained from an online panel discussions of our findings. Participants included men and women at different life stages and from a diverse range of ethnic backgrounds, and a more detailed account of the process is provided in the supplementary material. This study obtained ethics approval at City, University of London (ETH21220–299).

## Results

Of the weighted sample, we found 8.3% experienced workplace violence and 7.7% feared violence at work. As the unadjusted and adjusted estimates are similar, we focus discussion on the adjusted OR (OR_adj_) which account for the effect of the confounders, with the predicted probabilities presented in figure 1 and table S1 in the supplementary material. Workers in public administration and facilities had the highest risks of workplace violence, with a predicted probability of 0.138 (95% CI 0.116–0.160), followed by those working in health, residential care & social work at 0.118 (95% CI 0.103–0.133), though these categories were not statistically different from each other (see supplementary table S1 for all comparisons). The wholesale and retail sector and transportation and storage had the third and fourth highest predicted probability of violence. They were followed by education, accommodation and food services and arts, entertainment and other services. The lowest prevalence of violence was among workers in the manufacturing and construction sector, information, communication, finance and insurance sectors, and business administration and support services, with a predicted probability of around 0.040. Their risks were not significantly different from each other. Overall, the risk of violence varied between industrial sectors, though >1 in 10 workers were exposed to violence in the last 12 months in 30% of sectors, and >1 in 20 workers were exposed in 70% of sectors.

Fear of violence mimics violence risk in its distributions by sector, as shown in table 3 and figure 1 (see supplementary table S1 for all comparisons). People who worked in public administration and facilities were more likely to fear violence at work compared to all other sectors with a predicted probability of 0.124 (95% CI 0.103–0.145). Workers in the health/residential care and social work sectors had the second highest risks, 0.089 (95% CI 0.076–0.102), and this was statistically similar to the fears of those working in wholesale and retail, education, and accommodation and food services. In transportation and storage, manufacturing and construction, arts and entertainment, and business administration and support services sectors, 5–7% of workers feared violence at work.

Additionally, supplementary tables S3 and S4, the latter of which presents the full model, reveals an occupational gradient in exposure to violence at work, with the highest prevalence among the lower occupational classes, especially among service workers in shop and market sales, plant and machine operators and assemblers, and those working in elementary occupations. Similarly, the highest levels of fear of workplace violence were among elementary occupations and among service workers compared to the highest skilled occupational group.

### Effects and associations of workplace violence on common mental disorders

Prevalence of CMD in our sample was 20.3% at time t. Table 3 shows that of those who experienced violence in the workplace in the past 12 months, 34.1% reported CMD, and this was 39.4% among those who feared violence at work. The unadjusted and adjusted associations between violence and fear of violence are presented in table 3. Even when adjusting for CMD at t-1 and other confounders, we confirm that CMD were more common among people who had experienced violence at work compared to those who had not (OR_adj_ 1.400, 95% CI 1.182–1.658), as well as those who feared violence at work (OR_adj_ 2.103, 95% CI 1.779–2.487).

We also found the effect of violence to be persistent over time when we investigate CMD one year later at t+1 in table 4 (OR_adj_ 1.461, 95% CI 1.226–1.742 for violence at work; OR_adj_ 1.731, 95% CI 1.450–2.068 for fear of violence at work). Moreover, in these models, there was little evidence of strong difference in CMD risk by industrial sector or occupational status, suggesting a shared tendency by work type. Finally, we conducted sensitivity analyses of the functional form of our dependent variable, and found that a linear specification of CMD GHQ-12 led to the same conclusions (supplementary table S2).

### Lived experience engagement

Our statistical analyses are supplemented with the views of those with lived experience of violent victimization in support of trauma-informed practice, with additional details of our research protocol provided in the supplementary material. There was a unanimous sense from those with lived experience that our statistics on prevalence were likely to be the ‘tip of the iceberg’, given disclosure issues. It was noted how hard it was for someone who may be traumatized from their experience of violence at work to also be responsible for raising the issue with management, with the process of describing the incident frequently traumatizing in and of itself for the victim. People with lived experience reported that their managers were often annoyed or upset when presented with their accounts of what they experienced at work, and indeed managers were accused of minimizing or dismissing incidents reported, especially if they were between co-workers. Victim-survivors noted the significant mental health impacts of violence exposure and some noted that they feared being declared unfit for work should they disclose these to managers. Others stated that it was easier to report physical or ‘routine’ violent interactions, but that insidious bullying behaviors were much harder to report and prove. They stated a need for improved policies on how to deal with abusive customers or co-workers, yet others noted that in some sectors employees were told to expect violent incidents at work.

## Discussion

We deployed a data which to our knowledge has never previously been used to examine incidents of workplace violence, and one which is not currently used in official statistics on the topic. We had anticipated higher prevalence of workplace violence from the data we use here, the UKHLS, than that measured using the official crime data, with participants thought to be less likely to disclose workplace violence in a survey dedicated to the collection of criminal incidents (35). We found that 8.3% of employees reported having been threatened, insulted or physically attacked at work in the past 12 months. This prevalence rate is indeed higher than that established using official data, and though the indicator we use differs from official ones, as we included being insulted or shouted at in our conceptualization, our estimate offers useful insights into workplace violence that extend beyond criminal definitions (36).

We also sought to provide evidence of variance in workplace violence by industrial sector, given the current emphasis in the literature on sub-sectoral analyses, which we suggested risked creating a fragmented evidence base. We found, and in contradiction to current academic and policy discussion, evidence of workplace violence in all industrial sectors examined. Using a Standard Industrial Classification (SIC2007), we found 30% of the industrial sectors examined had high prevalence with predicted probabilities of workplace violence of ≥0.10, and 70% sectors had a predicted probability >0.05. We believe this finding underscores the importance of analyses of workplace violence at national levels and we hope it allows for a recalibration away from sub-sectoral analyses which risk generating the view that it is only certain sub-sectors of employee who face violence at work.

We measured prevalence of fear of workplace violence with a direct question asking respondents if they felt unsafe at work. We found 7.7% of employees reported feeling unsafe at work, and found fear of violence to closely mimic workplace violence risk. This is notable as fear of crime is often understood to be decoupled from risk of exposure to crime (25) and for this reason is sometimes treated as an illogical fear and disregarded as of little consequence. Yet, given the strong associations between fear of workplace violence and CMD risk, we believe fear of workplace violence needs to be better recognized as a significant problem for workers. Workplace violence also requires considerably better management, with systematic reviews noting a dearth of effective interventions which adhere to scientific protocols which would allow for assessments of efficacy (37). Current efforts appear primarily targeted at the health sector, and reviews frequently note limited evidence of decreases in workplace violence as a result of these initiatives (38) (39) (40). Furthermore, many interventions are targeted at front line staff only, rather than adopting a more effective systems wide approach (41).

There are few studies that deploy longitudinal data to the analysis of the effects of workplace violence on mental health and fewer still which use nationally representative prospective data. We add to the evidence base by providing an assessment of the long-term effects of workplace violence on mental health for a representative sample of UK employees. The questionnaire wording in the UKHLS already implies a temporal ordering, with questions on violent incidents and fear of violence, referencing the 12 months prior to interview, whereas the GHQ-12 battery asked about current well-being, and or how respondents had been feeling over the past few weeks. We further controlled for causal direction through the application of lagged variables of CMD one year prior to violence exposure, with mental health found to be predictive of both future exposure to violent incidents and current CMD risk. Furthermore, we also tested whether the effect of violence and fear of violence on CMD risk remained one year later, at t+1. All models found violence to be predictive of CMD risk, and in combination these suggest a casual pathway of workplace violence and fear of workplace violence on CMD risk. Future research might direct itself to examining variance in CMD risk by sector, which we were unable to provide here given our sample size.

Our findings suggest a pressing need for better enforcement of ILO convention No.190, which requires the prohibition in law of workplace violence and harassment and the sanction of those who inflict it (43), given the high prevalence rates of workplace violence and fear of violence established in many of the industrial sectors examined, alongside the prospective associations established between workplace violence and CMD. This need was echoed by our panel of experts with lived experience who noted the severe mental health effects of workplace violence as well as the considerable difficulties they faced when raising problematic behaviors to relevant managers at work. There is also a need for better research into effective interventions that reduce workplace violence (42), which would require longitudinal data to establish efficacy and deceased workplace violence over time. Future research should also consider the role of workers’ fear of violence and experiences of violence as well as the consequent CMD risks on productivity, which has to date not been examined at aggregate levels. Though panel data is typically ideal for the measurement of causal relationships, our study was somewhat limited by only having one wave of data on violence exposure and with little insight into the severity or frequency of violent incidents, as prior research has found a dose–response with heightened PTSD risk (20) depression risk (13) and suicide risk (12). Future research would also do well to examine differential effects by perpetrator type, with prior research suggesting that workplace violence between co-workers is more corrosive than between workers and service users (22).

Our final contribution was to examine the relative utility of targeted versus universal policy solutions to workplace violence. The problematic of workplace violence has been afforded enhanced political weight in the UK given its recent ratification of ILO convention no. 190. Though the convention seeks protections for all workers, in the UK legislative reform has sought to introduce targeted legislation for specific sectoral and occupational groups. These have included additional protections under the Assaults on Emergency Workers (Offences) Act 2018 & 2020, and most recently in April 2024 legislation is being proposed to enhance protections for retail workers. Yet, questions remain whether targeted legislation is appropriate and also whether the emphasis of legislation for sectoral sub-groups risks, again, fragmenting attempts to solve what may be a more universal problem than many recognize.

### Concluding remarks

We found exposure to workplace violence and fear of violence to increase the risk of CMD at baseline and one year after violence was reported. We also found high prevalence of workplace violence and fear of violence in many industrial sectors examined. We need better recognition of the extent to which workplace violence is experienced across multiple sectors and call for better systems wide interventions to mitigate the associated harms.

## Supplementary material

Supplementary material

## References

[1] ILO. Experiences of violence and harassment at work : A global first survey [Internet]. 2022. 56 p. Available from: https://www.ilo.org/wcmsp5/groups/public/---dgreports/---dcomm/documents/publication/wcms_863095.pdf

[2] Eurofound. Psychosocial risks to workers’ well-being: Lessons from the COVID-19 pandemic [Internet]. 2023. Available from: https://www.eurofound.europa.eu/en/publications/2023/psychosocial-risks-workers-well-being-lessons-covid-19-pandemic

[3] HSE. Violence at Work statistics, 2019. 2020;(March):1–14. Available from: https://www.hse.gov.uk/statistics/causinj/violence/index.htm

[4] Bunce A, Hashemi L, Clark C, Stansfeld S, Myers CA, McManus S. Prevalence and nature of workplace bullying and harassment and associations with mental health conditions in England: a cross-sectional probability sample survey. BMC Public Health 2024 Apr;24(1):1147. 10.1186/s12889-024-18614-7 38658961 PMC11044501

[5] Rasmussen CA, Hogh A, Andersen LP. Threats and physical violence in the workplace: a comparative study of four areas of human service work. J Interpers Violence 2013 Sep;28(13):2749–69. 10.1177/0886260513487987 23677967

[6] TUC. Domestic violence and the workplace a tuc survey report. 2014. Available from: Domestic_Violence_And_The_Workplace_0.pdf.

[7] Wu M, He Q, Imran M, Fu J. Workplace Bullying, Anxiety, and Job Performance: Choosing Between “Passive Resistance” or “Swallowing the Insult”? Front Psychol 2020 Jan;10:2953. 10.3389/fpsyg.2019.02953 32010020 PMC6978733

[8] van der Kolk B. Posttraumatic stress disorder and the nature of trauma. Dialogues Clin Neurosci 2000 Mar;2(1):7–22. 10.31887/DCNS.2000.2.1/bvdkolk 22034447 PMC3181584

[9] Balogh R, Gadeyne S, Vanroelen C, Warhurst C. Multidimensional employment trajectories and dynamic links with mental health: Evidence from the UK Household Longitudinal Study. Scand J Work Environ Health 2025 Jan;51(1):26–37. 10.5271/sjweh.4193 39476405 PMC11697615

[10] LanctÔt N. Guay S. The aftermath of workplace violence among healthcare workers: A systematic literature review of the consequences. Aggress Violent Behav 2014;19(5):492–501. 10.1016/j.avb.2014.07.010

[11] Piquero NL, Piquero AR, Craig JM, Clipper SJ. Assessing research on workplace violence, 2000-2012. Vol. 18. Aggress Violent Behav 2013;18(3):383–94. 10.1016/j.avb.2013.03.001

[12] Magnusson Hanson LL, Pentti J, Nordentoft M, Xu T, Rugulies R, Madsen IE et al. Association of workplace violence and bullying with later suicide risk: a multicohort study and meta-analysis of published data. Lancet Public Health 2023 Jul;8(7):e494–503. 10.1016/S2468-2667(23)00096-8 37393088

[13] Rudkjoebing LA, Hansen ÅM, Rugulies R, Kolstad H, Bonde JP. Exposure to workplace violence and threats and risk of depression: a prospective study. Scand J Work Environ Health 2021 Nov;47(8):582–90. 10.5271/sjweh.3976 34478560 PMC9058619

[14] Madsen IE, Svane-Petersen AC, Holm A, Burr H, Framke E, Melchior M et al. Work-related violence and depressive disorder among 955,573 employees followed for 6.99 million person-years. The Danish Work Life Course Cohort study: work-related violence and depression. J Affect Disord 2021 Jun;288:136–44. 10.1016/j.jad.2021.03.065 33887623

[15] Hogh A, Henriksson ME, Burr H. A 5-year follow-up study of aggression at work and psychological health. Int J Behav Med 2005;12(4):256–65. 10.1207/s15327558ijbm1204_6 16262544

[16] Magnavita N. The exploding spark: workplace violence in an infectious disease hospital--a longitudinal study. BioMed Res Int 2013;2013:316358. 10.1155/2013/316358 23936789 PMC3708405

[17] Guay S, Goncalves J, Jarvis J. A systematic review of exposure to physical violence across occupational domains according to victims’ sex. Aggress Violent Behav 2015;25:133–41. 10.1016/j.avb.2015.07.013

[18] Rudkjoebing LA, Bungum AB, Flachs EM, Eller NH, Borritz M, Aust B et al. Work-related exposure to violence or threats and risk of mental disorders and symptoms: a systematic review and meta-analysis. Scand J Work Environ Health 2020 Jul;46(4):339–49. 10.5271/sjweh.3877 31909816 PMC8506313

[19] Nyberg A, Kecklund G, Hanson LM, Rajaleid K. Workplace violence and health in human service industries: a systematic review of prospective and longitudinal studies. Occup Environ Med 2021 Feb;78(2):69–81. 10.1136/oemed-2020-106450 32414952 PMC7873420

[20] Pihl-Thingvad J, Andersen LL, Brandt LP, Elklit A. Are frequency and severity of workplace violence etiologic factors of posttraumatic stress disorder? A 1-year prospective study of 1,763 social educators. J Occup Health Psychol 2019 Oct;24(5):543–55. 10.1037/ocp0000148 31233309

[21] Andersen DR, Andersen LP, Gadegaard CA, Høgh A, Prieur A, Lund T. Burnout among Danish prison personnel: A question of quantitative and emotional demands. Scand J Public Health 2017 Dec;45(8):824–30. 10.1177/1403494817718644 28730915

[22] Serra-Sastre V. Workplace violence and intention to quit in the English NHS. Soc Sci Med 2024 Jan;340:116458. 10.1016/j.socscimed.2023.116458 38101172

[23] University of Essex I for S and EResearch. Understanding Society: Waves 1-13, 2009-2022 and Harmonised BHPS: Waves 1-18, 1991-2009. [data collection]. 18th Edition. UK Data Service. SN: 6614; 2023.

[24] Blom N, Gash V. Measures of Violence within the United Kingdom Household Longitudinal Survey and the Crime Survey for England and Wales: An Empirical Assessment. Soc Sci 2023 Dec;12(12).

[25] Noble J, Jardin A. From Victimization to Fear: Fear of Crime and its Variations among Victims. Br J Criminol 2020 Mar;60(2):468–89. 10.1093/bjc/azz051

[26] Goldberg DP, Gater R, Sartorius N, Ustun TB, Piccinelli M, Gureje O et al. The validity of two versions of the GHQ in the WHO study of mental illness in general health care. Psychol Med 1997 Jan;27(1):191–7. 10.1017/S0033291796004242 9122299

[27] Anjara SG, Bonetto C, Van Bortel T, Brayne C. Using the GHQ-12 to screen for mental health problems among primary care patients: psychometrics and practical considerations. Int J Ment Health Syst 2020 Aug;14(1):62. 10.1186/s13033-020-00397-0 32793301 PMC7418321

[28] Pierce M, Hope H, Ford T, Hatch S, Hotopf M, John A et al. Mental health before and during the COVID-19 pandemic: a longitudinal probability sample survey of the UK population. Lancet Psychiatry 2020 Oct;7(10):883–92. 10.1016/S2215-0366(20)30308-4 32707037 PMC7373389

[29] Rönnblad T, Grönholm E, Jonsson J, Koranyi I, Orellana C, Kreshpaj B et al. Precarious employment and mental health: a systematic review and meta-analysis of longitudinal studies. Scand J Work Environ Health 2019 Sep;45(5):429–43. 10.5271/sjweh.3797 31165899

[30] Golu F. Predictors of Domestic Violence – Comparative Analysis. Procedia Soc Behav Sci 2014 Apr;127:611–5. 10.1016/j.sbspro.2014.03.321

[31] Gash V, Mertens A, Gordo LR. Are fixed-term jobs bad for your health?: A comparison of West-Germany and Spain. Eur Soc 2007;9(3): 10.1080/14616690701314150

[32] Kvart S, Jonsson J, Bodin T, Håkansta C, Kreshpaj B, Orellana C et al. Precarious employment and psychosocial hazards: A cross-sectional study in stockholm county. Int J Environ Res Public Health 2021 Oct;18(21):11218. 10.3390/ijerph182111218 34769737 PMC8582981

[33] Wilkins AS. To lag or not to lag?:Re-evaluating the use of lagged dependent variables in regression analysis. Vol. 6, Political Science Research and Methods. Cambridge University Press; 2018. p. 393–411.

[34] Lynn P, Kaminska O. Weighting Strategy for Understanding Society Understanding Society Working Paper Series [Internet]. 2010. Available from: www.understandingsociety.org.uk/overview/governance/

[35] Jones T, Robinson A, Fevre R, Lewis D. Workplace assaults in Britain: understanding the influence of individual and workplace characteristics. Br J Criminol 2011 Jan;51(1):159–78. 10.1093/bjc/azq064

[36] Davies E, Obolenskaya P, Francis B, Blom N, Phoenix J, Pullerits M et al. Definition and Measurement of Violence in the Crime Survey for England and Wales: Implications for the Amount and Gendering of Violence. Br J Criminol 2024 Jul;•••: 10.1093/bjc/azae050

[37] Anderson L, FitzGerald M, Luck L. An integrative literature review of interventions to reduce violence against emergency department nurses. J Clin Nurs 2010 Sep;19(17-18):2520–30. 10.1111/j.1365-2702.2009.03144.x 20553349

[38] Aust B, Møller JL, Nordentoft M, Frydendall KB, Bengtsen E, Jensen AB et al. How effective are organizational-level interventions in improving the psychosocial work environment, health, and retention of workers? A systematic overview of systematic reviews. Scand J Work Environ Health 2023 Jul;49(5):315–29. 10.5271/sjweh.4097 37158211 PMC10713994

[39] Spelten E, Thomas B, O’Meara PF, Maguire BJ, FitzGerald D, Begg SJ. Organisational interventions for preventing and minimising aggression directed towards healthcare workers by patients and patient advocates. Vol. 2020, Cochrane Database of Systematic Reviews. John Wiley and Sons Ltd; 2020. 10.1002/14651858.CD012662.pub2 PMC7197696 32352565

[40] Baby M, Gale C, Swain N. Communication skills training in the management of patient aggression and violence in healthcare. Aggress Violent Behav 2018;39:67–82. 10.1016/j.avb.2018.02.004

[41] Sheppard DM, Newnam S, Louis RM, Perrett MS. Factors contributing to work-related violence: A systematic review and systems perspective. Saf Sci 2022 Oct;154: 10.1016/j.ssci.2022.105859

[42] Wassell JT. Workplace violence intervention effectiveness: A systematic literature review. Vol. 47. Saf Sci 2009;47(8):1049–55. 10.1016/j.ssci.2008.12.001

[43] International Labour Organisation. Violence and Harassment in the World of Work A Guide on Convention No. 190 and Recommendation No. 206. International Labour Organisation (ILO); 2021.

