# Supplementary material for: Workplace violence and fear of violence: an assessment of prevalence across industrial sectors and its mental health effects
Source: Scand J Work Environ Health. 2025 Aug 29;51(5):370–9. doi: 10.5271/sjweh.4230 (PMC12416159; doi:10.5271/sjweh.4230)
Supplement: Supplementary material [file SJWEH-51-370-S001.pdf]

Workplace violence and fear of violence: an assessment of prevalence across industrial sectors and its mental health effects[1]

by Vanessa Gash, DPhil,[2] Niels Blom, PhD

1. Supplementary material
2. Correspondence to: Vanessa Gash, City St Georges, University of London, London, UK. [E-mail: Vanessa.Gash.1@citystgeorges.ac.uk]

Lived Experience Engagement

A key component of the research agenda of the VISION consortium concerns the active inclusion of those with lived experience of violence, including domestic violence, sexual violence and workplace violence. We use the research involvement framework developed by UK based charity 'Survivors Voices' which seeks to improve trauma response by embedding the views and experiences of survivors of abuse in the research process. For this paper, we obtained lived experience engagement in the following way. A meeting was set up by the director of the Violence, Abuse and Mental Health Network (VAMHN), Dr Sian Oram, a Reader in Women's Mental Health at the Institute of Psychiatry, Psychology & Neuroscience at King's College London and members of the Lived Experience Action Group (LEAG) a group of individuals with experience of violence or abuse and associated mental health difficulties. The meeting was held online in January 2024, with Dr Gash presenting her findings on the 'impact of violence and abuse (including intimate partner violence) on employment outcomes' to a panel of nine with lived experience. The panel was composed of men and women, young and old, and included those with minority ethnic backgrounds, as well as LGBTQ+ respondents. The panel of those with lived experience had been forwarded presentation slides alongside proposed questions for collective discussion prior to the meeting. Those questions were:

(Q1) Were the results as you expected? Did anything surprise or particularly interest you?

(Q2) If and when would people be willing to disclose their experience of violence at work to HR/managers?

(Q3) What could/should employers do to keep people who experience violence at work employed?

(Q4) If and when would people be willing to disclose their experience with intimate partner violence to HR/managers?

(Q5) What could/should employers do to keep people who experience intimate partner abuse employed?

(Q6) What do you think might be the long-term effects of violence and abuse on whether and how people are able to work?

(Q7) How might people be better supported?

Discussion occurred in the format of an online focus group over a two hour-period and the meeting was not video or voice recorded to protect and preserve anonymity. Minutes of the discussion were taken instead. A draft summary of the discussion was also forwarded to the panel for verification.

Table S1. Predicted probability of workplace violence and fear of violence at work by industrial sector, including pair-wise comparisons, based on adjusted models from table 2, N=11,366 [CI=confidence interval].

Panel 1: Workplace Violence by industrial classification

		Difference in predicted probability, p values									
	Predicted probability	*Industrial classification*									
Industrial classification	(95% CI)	(1)	(2)	(3)	(4)	(5)	(6)	(7)	(8)	(9)	(10)
(1) Public administration & facilities	0.138 (0.116 - 0.160)		0.132	0.006	0.000	0.000	0.000	0.000	0.000	0.000	0.000
(2) Health, residential care & social work	0.118 (0.103 - 0.133)	0.132		0.108	0.008	0.003	0.000	0.000	0.000	0.000	0.000
(3) Wholesale & retail	0.100 (0.086 - 0.115)	0.006	0.108		0.316	0.059	0.013	0.017	0.000	0.000	0.000
(4) Education	0.089 (0.072 - 0.105)	0.000	0.008	0.316		0.339	0.137	0.163	0.000	0.000	0.000
(5) Transportation & storage	0.074 (0.052 - 0.097)	0.000	0.003	0.059	0.339		0.729	0.718	0.033	0.045	0.010
(6) Arts, entertainment and other services	0.069 (0.049 - 0.089)	0.000	0.000	0.013	0.137	0.729		0.977	0.056	0.074	0.022
(7) Accommodation & food services	0.069 (0.046 - 0.091)	0.000	0.000	0.017	0.163	0.718	0.977		0.090	0.108	0.043
(8) Business administration & support services	0.046 (0.034 - 0.058)	0.000	0.000	0.000	0.000	0.033	0.056	0.090		0.961	0.638
(9) Information, communication, finance & insurance	0.046 (0.030 - 0.061)	0.000	0.000	0.000	0.000	0.045	0.074	0.108	0.961		0.730
(10) Manufacturing & construction	0.043 (0.032 - 0.053)	0.000	0.000	0.000	0.000	0.010	0.022	0.043	0.638	0.730	

Panel 2: Fear of violence at work by industrial classification

		Difference in predicted probability, p values									
	Predicted probability	*Industrial classification*									
Industrial classification	(95% CI)	(1)	(2)	(3)	(4)	(5)	(6)	(7)	(8)	(9)	(10)
(1) Public administration & facilities	0.124 (0.103 - 0.145)		0.005	0.001	0.000	0.000	0.000	0.003	0.000	0.000	0.000
(2) Health, residential care & social work	0.089 (0.076 - 0.102)	0.005		0.496	0.142	0.008	0.006	0.352	0.015	0.000	0.031
(3) Wholesale & retail	0.082 (0.069 - 0.096)	0.001	0.496		0.461	0.028	0.032	0.653	0.083	0.004	0.116
(4) Education	0.075 (0.060 - 0.089)	0.000	0.142	0.461		0.139	0.151	0.899	0.332	0.031	0.435
(5) Transportation & storage	0.056 (0.036 - 0.075)	0.000	0.008	0.028	0.139		0.886	0.167	0.459	0.671	0.372
(6) Arts, entertainment and other services	0.058 (0.039 - 0.076)	0.000	0.006	0.032	0.151	0.886		0.196	0.543	0.548	0.467
(7) Accommodation & food services	0.077 (0.054 - 0.099)	0.003	0.352	0.653	0.899	0.167	0.196		0.385	0.068	0.457
(8) Business administration & support services	0.065 (0.051 - 0.079)	0.000	0.015	0.083	0.332	0.459	0.543	0.385		0.184	0.880
(9) Information, communication, finance & insurance	0.050 (0.033 - 0.067)	0.000	0.000	0.004	0.031	0.671	0.548	0.068	0.184		0.150
(10) Manufacturing & construction	0.066 (0.052 - 0.081)	0.000	0.031	0.116	0.435	0.372	0.467	0.457	0.880	0.150	

Table S2. Results from the linear regression analysis, showing the showing the association of having common mental disorders (GHQ-12) at time t and t +1 by violence at work, fear of violence at work, and industrial classification [CI=confidence interval].

	Common Mental Disorders at T (N=11,366)		Common Mental Disorders at T + 1 (N=9,908)	
	Unadjusted B (95% CI)	Adjusted B (95% CI) [a]	Unadjusted B (95% CI)	Adjusted B (95% CI) [a]
Common Mental Disorders at t-1				
No	1	1	1	1
Yes	0.457 (0.439 - 0.475)	0.429 (0.411 - 0.447)	0.406 (0.386 - 0.427)	0.374 (0.353 - 0.395)
Violence at work				
No	1	1	1	1
Yes	1.352 (1.150 - 1.555)	0.502 (0.305 - 0.699)	1.270 (1.042 - 1.498)	0.528 (0.300 - 0.757)
Fear of violence at work				
No	1	1	1	1
Yes	1.747 (1.539 - 1.956)	0.993 (0.790 - 1.196)	1.444 (1.206 - 1.681)	0.670 (0.434 - 0.907)
Industrial classification				
Public administration & facilities	1	1	1	1
Health, residential care & social work	-0.100 (-0.330 - 0.130)	-0.168 (-0.381 - 0.044)	-0.127 (-0.379 - 0.125)	-0.225 (-0.464 - 0.015)
Education	-0.051 (-0.295 - 0.192)	-0.022 (-0.249 - 0.206)	-0.036 (-0.302 - 0.231)	-0.032 (-0.288 - 0.225)
Wholesale & retail	-0.142 (-0.389 - 0.104)	-0.059 (-0.289 - 0.171)	-0.068 (-0.339 - 0.203)	0.004 (-0.256 - 0.264)
Transportation & storage	-0.771 (-1.102 - -0.441)	-0.164 (-0.482 - 0.155)	-0.705 (-1.076 - -0.334)	-0.087 (-0.454 - 0.281)
Manufacturing & construction	-0.728 (-0.965 - -0.492)	-0.263 (-0.491 - -0.035)	-0.716 (-0.977 - -0.454)	-0.225 (-0.485 - 0.034)
Info., communication, finance & insurance	-0.253 (-0.532 - 0.026)	-0.039 (-0.293 - 0.215)	-0.417 (-0.722 - -0.113)	-0.211 (-0.496 - 0.074)
Business administration & support services	-0.209 (-0.456 - 0.038)	0.011 (-0.214 - 0.237)	-0.204 (-0.475 - 0.068)	-0.028 (-0.283 - 0.226)
Arts, entertainment and other services	-0.082 (-0.389 - 0.225)	0.020 (-0.261 - 0.301)	-0.047 (-0.388 - 0.295)	0.029 (-0.292 - 0.350)
Accommodation & food services	-0.096 (-0.455 - 0.264)	-0.350 (-0.687 - -0.013)	0.232 (-0.176 - 0.641)	-0.077 (-0.470 - 0.317)

[a] Adjusted for occupational classification, contract type, work hours, working time, work authority, gender, age, and ethnicity.

Table S3. Results from the logistic regression analysis, showing the risk of violence at work by industrial classification N=11,366. [OR=odds ratio; CI=confidence interval; AOR=adjusted odds ratio]

	Number of observations	Observations violence (%)	Violence at work OR (95% CI)	AOR (95% CI)
Industrial classification				
Public administration & facilities	1086	125 (12.9)	1	1
Health, residential care & social work	2167	262 (12.3)	0.944 (0.753 - 1.183)	0.828 (0.651 - 1.054)
Education	1534	118 (7.9)	0.579 (0.446 - 0.752)	0.597 (0.451 - 0.792)
Wholesale & retail	1306	146 (12.1)	0.926 (0.726 - 1.180)	0.688 (0.530 - 0.894)
Transportation & storage	498	43 (9.3)	0.692 (0.485 - 0.989)	0.491 (0.330 - 0.731)
Manufacturing & construction	1647	62 (4.0)	0.280 (0.207 - 0.379)	0.268 (0.192 - 0.374)
Information, communication, finance & insurance	861	37 (3.8)	0.262 (0.176 - 0.390)	0.291 (0.194 - 0.436)
Business administration & support services	1341	54 (3.9)	0.276 (0.199 - 0.383)	0.294 (0.210 - 0.411)
Arts, entertainment and other services	567	37 (7.2)	0.521 (0.364 - 0.745)	0.451 (0.312 - 0.654)
Accommodation & food services	359	30 (9.4)	0.695 (0.470 - 1.026)	0.448 (0.296 - 0.678)
Occupational classification				
Legislators, senior officials & managers	1767	116 (6.6)	1	1
Professionals	2023	144 (7.2)	1.088 (0.842 - 1.404)	0.905 (0.683 - 1.198)
Technicians & associate professionals	2096	158 (7.3)	1.112 (0.865 - 1.430)	0.816 (0.624 - 1.067)
Clerks	1486	105 (7.5)	1.149 (0.876 - 1.507)	0.956 (0.716 - 1.275)
Service workers, shop & market sales	1846	235 (14.0)	2.298 (1.827 - 2.891)	1.189 (0.921 - 1.535)
Agricultural, fishery, craft, & related trades	692	38 (5.9)	0.892 (0.625 - 1.272)	1.095 (0.745 - 1.609)
Plant & machine operators & assemblers	583	49 (7.6)	1.161 (0.814 - 1.655)	0.872 (0.586 - 1.299)
Elementary occupations	873	69 (8.7)	1.340 (1.002 - 1.791)	0.979 (0.716 - 1.340)
Contract type				
Permanent	10550	851 (8.3)	1	1
Temporary	816	63 (8.9)	1.081 (0.843 - 1.388)	1.135 (0.876 - 1.471)
Work hours				
Fulltime	8395	718 (8.7)	1	1
Parttime	2971	196 (7.3)	0.830 (0.710 - 0.971)	0.637 (0.535 - 0.759)
Working time				
Standard	7472	410 (5.6)	1	1
Nonstandard	3894	504 (13.2)	2.560 (2.237 - 2.929)	2.169 (1.873 - 2.511)
Work autonomy [a]			0.634 (0.586 - 0.687)	0.714 (0.654 - 0.780)

Table continues on next page

Table S3. Continued.

	Number of observations	Observations violence (%)	Violence at work OR (95% CI)	AOR (95% CI)
Gender				
Men	5136	371 (7.5)	1	1
Women	6230	543 (9.2)	1.254 (1.096 - 1.433)	1.062 (0.906 - 1.246)
Age [b]			0.987 (0.982 - 0.992)	0.991 (0.986 - 0.997)
Ethnicity				
White (British)	9839	804 (8.3)	1	1
Mixed, multiple or other	282	25 (9.6)	1.172 (0.749 - 1.835)	1.179 (0.744 - 1.867)
Asian (British)	879	53 (8.6)	1.041 (0.740 - 1.466)	0.969 (0.682 - 1.377)
Black (British)	366	32 (9.1)	1.110 (0.695 - 1.772)	0.877 (0.542 - 1.419)
Constant				0.359 (0.233 - 0.552)

[a] Increase in OR/AOR by 1 on scale ranging from 0–3

[b] Increase in OR/AOR by 1 on scale ranging from 17–85

Table S4. Results from the logistic regression analysis, showing the risk of workplace violence and fear of violence by industrial classification N=13,538. [OR=odds ratio; CI=confidence interval; AOR=adjusted odds ratio]

	Number of observations	Observations fearing violence (%)	Fear of violence at work	
			OR (95% CI)	AOR (95% CI)
Industrial classification				
Public administration & facilities	1086	113 (11.2)	1	1
Health, residential care & social work	2167	208 (9.6)	0.842 (0.659 - 1.075)	0.678 (0.524 - 0.878)
Education	1534	115 (7.4)	0.634 (0.482 - 0.833)	0.559 (0.418 - 0.749)
Wholesale & retail	1306	119 (10.2)	0.895 (0.690 - 1.160)	0.623 (0.471 - 0.824)
Transportation & storage	498	33 (6.7)	0.564 (0.375 - 0.846)	0.405 (0.261 - 0.629)
Manufacturing & construction	1647	70 (5.4)	0.447 (0.336 - 0.595)	0.490 (0.357 - 0.673)
Information, communication, finance & insurance	861	30 (3.6)	0.299 (0.198 - 0.450)	0.360 (0.238 - 0.546)
Business administration & support services	1341	55 (5.6)	0.467 (0.346 - 0.631)	0.478 (0.350 - 0.651)
Arts, entertainment and other services	567	24 (6.0)	0.505 (0.343 - 0.744)	0.421 (0.282 - 0.627)
Accommodation & food services	359	38 (12.3)	1.103 (0.768 - 1.585)	0.574 (0.389 - 0.848)
Occupational classification				
Legislators, senior officials & managers	1767	77 (4.5)	1	1
Professionals	2023	126 (6.7)	1.518 (1.139 - 2.024)	1.340 (0.986 - 1.822)
Technicians & associate professionals	2096	130 (5.9)	1.322 (0.988 - 1.769)	1.053 (0.777 - 1.426)
Clerks	1486	101 (7.2)	1.652 (1.223 - 2.231)	1.334 (0.975 - 1.826)
Service workers, shop & market sales	1846	225 (13.2)	3.228 (2.482 - 4.197)	1.927 (1.447 - 2.565)
Agricultural, fishery, craft, & related trades	692	29 (5.3)	1.191 (0.807 - 1.759)	1.296 (0.856 - 1.963)
Plant & machine operators & assemblers	583	36 (6.6)	1.500 (1.014 - 2.220)	1.357 (0.887 - 2.076)
Elementary occupations	873	81 (12.3)	2.968 (2.211 - 3.984)	2.348 (1.716 - 3.214)
Contract type				
Permanent	10550	741 (7.6)	1	1
Temporary	816	64 (8.9)	1.176 (0.916 - 1.512)	1.149 (0.887 - 1.488)
Work hours				
Fulltime	8395	566 (7.4)	1	1
Parttime	2971	239 (8.6)	1.170 (1.005 - 1.361)	0.876 (0.739 - 1.040)
Working time				
Standard	7472	391 (5.7)	1	1
Nonstandard	3894	414 (11.4)	2.153 (1.875 - 2.473)	1.797 (1.545 - 2.090)
Work autonomy [a]			0.669 (0.616 - 0.726)	0.798 (0.728 - 0.874)

Table continues on next page

Table S4. Continued.

	Number of observations	Observations fearing violence (%)	Fear of violence at work OR (95% CI)	AOR (95% CI)
Gender				
Men	5136	281 (6.3)	1	1
Women	6230	524 (9.0)	1.474 (1.281 - 1.697)	1.314 (1.112 - 1.553)
Age [b]			0.983 (0.978 - 0.988)	0.987 (0.981 - 0.992)
Ethnicity				
White (British)	9839	686 (7.7)	1	1
Mixed, multiple or other	282	26 (9.6)	1.270 (0.811 - 1.989)	1.233 (0.780 - 1.949)
Asian (British)	879	56 (6.0)	0.771 (0.517 - 1.151)	0.730 (0.485 - 1.098)
Black (British)	366	37 (11.3)	1.524 (0.994 - 2.337)	1.282 (0.828 - 1.985)
Constant				0.197 (0.125 - 0.312)

[a] Increase in OR/AOR by 1 on scale ranging from 0–3

[b] Increase in OR/AOR by 1 on scale ranging from 17–85

Table S5. Selecting only respondents who participated before the first Covid-19 lockdown in the United Kingdom. Results from the logistic regression analysis, showing the risk of workplace violence and fear of violence at work by industrial classification, and their associations with Common Mental Health disorders N= 7,799. [OR=odds ratio; CI=confidence interval; AOR=adjusted odds ratio]

	Violence at work AOR (95% CI) [a]	Fear of violence at work AOR (95% CI) [a]	CMD at t AOR (95% CI) [a]
Common Mental Disorders at t-1			
No			
Yes			5.327 (4.655 - 6.097)
Violence at work			
No			
Yes			1.457 (1.184 - 1.793)
Fear of violence at work			
No			
Yes			2.023 (1.611 - 2.539)
Industrial classification			
Public administration & facilities			
Health, residential care & social work	0.871 (0.657 - 1.157)	0.728 (0.525 - 1.007)	1.038 (0.809 - 1.332)
Education	0.669 (0.484 - 0.925)	0.490 (0.333 - 0.720)	1.040 (0.795 - 1.361)
Wholesale & retail	0.703 (0.515 - 0.961)	0.530 (0.369 - 0.761)	1.136 (0.866 - 1.491)
Transportation & storage	0.542 (0.346 - 0.852)	0.454 (0.272 - 0.758)	0.948 (0.636 - 1.411)
Manufacturing & construction	0.251 (0.168 - 0.374)	0.294 (0.188 - 0.462)	0.762 (0.572 - 1.015)
Information, communication, finance & insurance	0.268 (0.164 - 0.437)	0.244 (0.135 - 0.443)	1.034 (0.761 - 1.403)
Business administration & support services	0.341 (0.234 - 0.497)	0.470 (0.318 - 0.694)	1.061 (0.810 - 1.390)
Arts, entertainment and other services	0.476 (0.310 - 0.731)	0.488 (0.304 - 0.782)	0.989 (0.708 - 1.380)
Accommodation & food services	0.418 (0.248 - 0.705)	0.441 (0.263 - 0.737)	0.710 (0.464 - 1.086)

[a] Adjusted for occupational classification, contract type, work hours, working time, work autonomy, gender, age, and ethnicity.
